# Necrology

**Published:** 1899-08

**Authors:** 


### NECROLOGY.

### MICHAEL J. TREACY, M.R.C.V.S.

DR. TREACY, late veterinarian Eighth U. S. Cavalry, died of yellow fever at Puerto Principe, Cuba, July 15, 1899. Dr. Treacy was a graduate of the Royal College at Glasgow, class of 1874, a part of his time having been spent at the Edinburgh school. He entered the U. S. Army as veterinary surgeon of the Seventh U. S. Cavalry, April 12, 1883, and served in that regiment until October 15, 1887, when he resigned to enter civil practice. He reëntered the service of U. S. Eighth Cavalry, January 7, 1889. Thus he served his country in a most faithful manner for fifteen years. During this long period he was a hard worker in a most thankless position. A veterinarian of ability, a splendid operator, a contributor of many articles of real merit to the professional and military journals, his writings in the latter showing him to be a military veterinarian of the highest order. By a strange coincidence his death occurred the same day that the Army Board of Examiners passed him to the grade of first-class veterinarian, with the pay and allowances of an officer, he having made a high mark before the Board. Since his entrance into the service Dr. Treacy was the leader in the fight for rank for army veterinarians, as his many articles in the journals and papers will attest. His work won for him praise from the entire army and the thorough respect of all officers he served with, and being a man of ability, he always commanded the respect and consideration of officers in command.

Dr. Treacy was a brave man. He went to Cuba to do his duty without any hope of glory, promotion, or even the small pittance of a pension accorded to every private in the army. When a man leaves his family and goes to "pest-ridden" Cuba under such conditions he is of the heroic order, which requires more real courage than the mere facing of the bullets of a cowardly, retreating enemy. Such a man was Dr. Treacy. Those who were in touch with his life and doings say that for three months past he played the part of a martyr in his love for the profession of his choice; though sick for months with Cuban fever, he would work continuously, doing double duty all the time, for the climate has almost ruined American cavalry horses, and his daily sick-reports show the prodigious amount of work he has done, despite his weakened condition.

Dr. Treacy was about fifty years of age. He leaves a widow, who only recently returned from Cuba, where she had faithfully nursed the doctor through a six-weeks' attack of Cuban fever. Dr. Treacy was an active member of the American Veterinary Association.

---

State Veterinarian Lovejoy seems to be having a serious time with the dairy-owers of McHenry County, Illinois, in endeavoring to eliminate tuberculosis from their herds. The extent of the disease has been found to exceed all anticipation in this direction, another evidence of the destructive nature of this disease when it once gains a foothold. These same protesting farmers will, in a few years, be seeking the help and assistance the State is desirous of giving when a campaign of education brings the fruits of a better understanding.

